# Rethinking Lockdown Policies in the Pre-Vaccine Era of COVID-19: A Configurational Perspective

**DOI:** 10.3390/ijerph19127142

**Published:** 2022-06-10

**Authors:** Ziang Zhang, Chao Liu, Robin Nunkoo, Vivek A. Sunnassee, Xiaoyan Chen

**Affiliations:** 1 School of Geography, Nanjing Normal University, Nanjing 210023, China; zhangzang5@mail2.sysu.edu.cn; 2 Faculty of Hospitality & Tourism Management, Macau University of Science and Technology, Taipa, Macau 999078, China; 3 Department of Management, University of Mauritius, Reduit MU 80837, Mauritius; r.nunkoo@uom.ac.mu; 4 School of Tourism and Hospitality, University of Johannesburg, P.O. Box 524, Auckland Park 2006, South Africa; 5 Griffith Institute for Tourism, Griffith University, Gold Coast, QLD 4222, Australia; 6 Copenhagen Business School, Porcelaenshaven 18A, DK-2000 Frederiksberg, Denmark; 7 Westminster Business School, University of Westminster, 35 Marylebone Road, London NW1 5LS, UK; v.v.sunnassee@westminster.ac.uk; 8 School of Humanities, Jiangsu University of Technology, Changzhou 213001, China; cxy220@163.com

**Keywords:** lockdown policy, high epidemic, pandemic, COVID-19, fsQCA, comparative policy analysis

## Abstract

The significance of lockdown policies for controlling the COVID-19 pandemic is widely recognized. However, most studies have focused on individual lockdown measures. The effectiveness of lockdown policy combinations has not been examined from a configurational perspective. This research applies fuzzy-set qualitative comparative analysis (fsQCA) to examine different lockdown policy combinations associated with high-epidemic situations in 84 countries. A high-epidemic situation can occur through three different “weak-confined” patterns of lockdown policy combinations. The findings demonstrate that a combination of lockdown policies is more successful than any single lockdown policy, whereas the absence of several key measures in policy combinations can lead to a high-epidemic situation. The importance of international travel controls can become obscured when they are the only measures adopted, and a high-epidemic situation can still arise where restrictions are placed on international travel but not on public transport or when workplaces are closed but schools remain open.

## 1. Introduction

The first cluster of COVID-19 cases was discovered in December 2019 in Wuhan, China. By 2020, the pandemic had spread rapidly to several other countries. The World Health Organisation declared COVID-19 a public health emergency on 30 January 2020 and a pandemic on 11 March 2020. As of 10 January 2022, there were 2,236,613 daily new cases, which rose to rose to 3,372,994 on 13 January 2022. Over the same three days, daily deaths increased from 5448 to 8168 (https://www.worldometers.info/coronavirus/, accessed on 15 January 2022). With no vaccine and limited pharmaceutical interventions to treat COVID-19 patients in 2020 [1,2], governments relied on non-pharmaceutical interventions (NPIs), such as lockdowns involving school, workplace, and transport closure; restrictions on internal movement; and international travel restrictions to control the pandemic [3]. It was not until December 2020 that COVID-19 vaccines became available. However, vaccine shortages and equitable distribution have been important challenges that have plagued countries around the world. Many countries are unable to meet the minimum vaccination targets set by the WHO [4,5]. At the same time, virus variants also pose a challenge for vaccine effectiveness. Studies have shown that novel variants pose a significant challenge to the clinical effectiveness of currently available vaccines and therapeutic antibodies [6,7,8]. In addition, there are also differences in the willingness of people in different countries to get vaccinated [9,10,11,12]. Vaccine hesitancy has become another threat to global health in the post-vaccine era. A degree of public reluctance to being vaccinated and perceptions of the effectiveness of the different vaccines have also hindered vaccination programs worldwide [13].

Consequently, even with the availability of vaccines, the significance of NPIs has been constantly reiterated by governments, public health experts, and medical practitioners [14,15]. The aim of NPIs is to reduce the spread of the virus by limiting human-to-human contact [16,17] through social distancing [18] and lockdown measures [19]. However, it is difficult to assess the effectiveness of such measures in containing the pandemic for the following reasons. First, different countries implemented different combinations of NPIs to control the pandemic. For example, Singapore combined strong testing efforts with home isolation policies [20], whereas China employed a combination of emergency controls and strict lockdowns in high-risk regions [21,22]. Second, the level of stringency of such measures also differed across nations. For example, as of June 2020, while countries such as Yemen, Mexico, India, Germany, and the United States implemented full school closures, other countries, such as Japan and China, were less stringent on this policy. Some nations also relied on full border closures, whereas others only partially closed their borders to restrict travel. Third, there are socio-cultural, economic, political, and health attributes that influence COVID-19 cases and deaths that differ across nations [1,23,24]. For example, whereas sanitary measures such as the wearing of a face mask have been the norm in Southeast Asian countries since the outset of the pandemic, uptake has been slow in other nations.

Several studies have examined the significance of NPIs, highlighting their importance in controlling the pandemic [18,25,26,27]. However, two research limitations can be identified. First, at the theoretical level, studies have mainly focused on individual lockdown measures [28,29,30] instead of adopting an integrated/configurational perspective that analyses the inter-relationships between various NPIs and pandemic spread. Second, at the methodological level, these studies tend to explore the “net effect” of specific factors but ignore the “combined effects” of multiple related factors on COVID-19 contamination.

Therefore, the previously published literature offers limited insights into the reality that the impact of lockdown policies on a pandemic is not only determined by individual lockdown policies but that it also depends on the combined effect of different policy combinations. This problem of causal complexity cannot be fully explained using traditional regression analysis, which can only explore the net effect of a single factor or the moderating effect of up to three variables [31]. There is therefore a need for a more insightful understanding of the roles of different lockdown policies in mitigating the spread of COVID-19 in order to assess their theoretical and practical importance [32]. The complex nature of lockdown policies and their influence on pandemic spread can be fully understood by studying the “combined effects” of multiple factors. To address these gaps, in this paper, we adopt a configurational perspective, which is a new way of thinking that attempts to decipher the causal complexity that underpins social and organizational phenomena [33]. This study uses fuzzy-set qualitative comparative analysis (fsQCA) to examine the conjectural causation and interactive relationships among the various lockdown measures that countries have implemented [34,35]. FsQCA has been widely used in research that addresses public policy issues [36,37,38,39] and in comparative policy studies [40]. We utilise the fsQCA method to study the response effect, action path, and key patterns of lockdown policies in high-epidemic countries from a configurational perspective. We use data from the COVID-19 Government Response Tracker developed by the University of Oxford, UK [3].

This research advances the current literature in the following ways: (i) It explores different lockdown policy combinations in high-epidemic countries in the pre-vaccine period. In so doing, we extract representative patterns from these policy pathways based on their respective core conditions. (ii) It exposes the complexity of the inter-relationships among different policies through an examination of the interactions between them rather than by focusing on the independent net effect of a single lockdown policy on the occurrence of a high-pandemic situation. (iii) It contributes empirical evidence to research on public health and governance through a macro-comparative investigation of lockdown policies across various countries. Practically, this research provides valuable insights into the effectiveness of NPIs in the control of COVID-19. The findings can also be used to manage future pandemics that require lockdown policies.

## 2. COVID-19 Lockdown Policies

**Figure 1:**
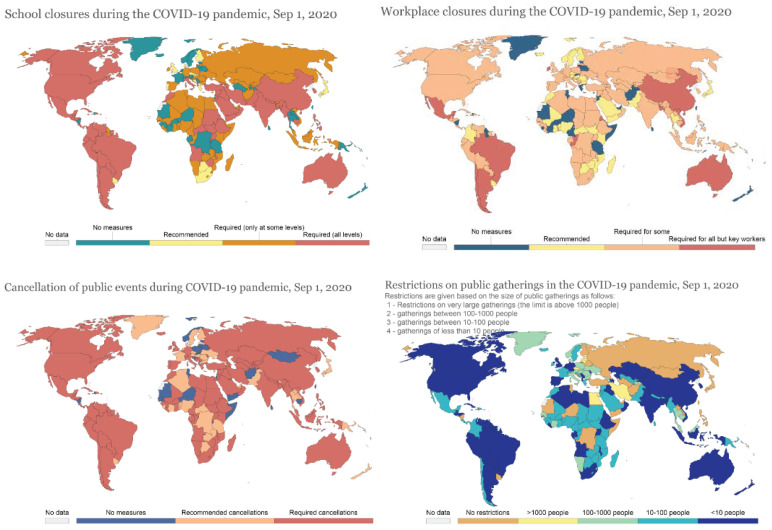

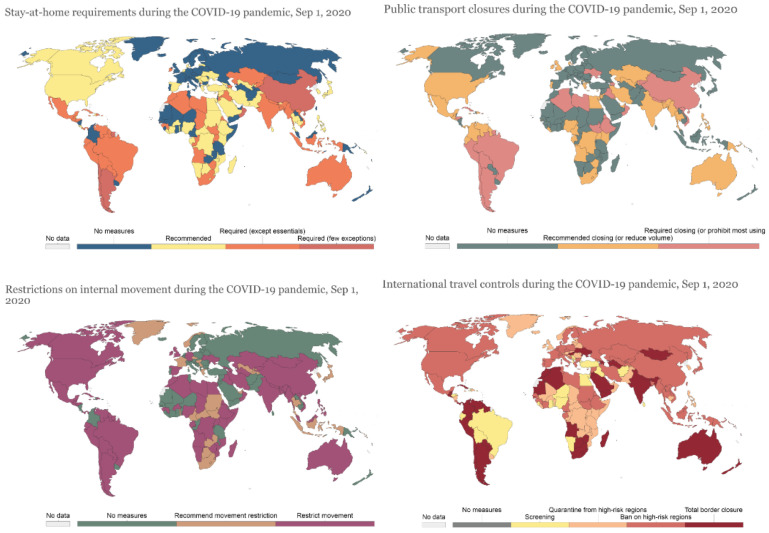
Global overview of the eight lockdown policies in the pre-vaccine era (1 September 2020) (Source: https://ourworldindata.org/policy-responses-covid, accessed on 15 January 2022).

Lockdown policies have been a key response in the fight against COVID-19. During the early weeks of the pandemic, the city of Wuhan, China, initiated a travel restriction order banning the unauthorised movement of people to and from the city [41,42,43]. Many other countries implemented various sets of NPIs to contain the pandemic. In order to provide an intuitive understanding of the heterogeneity of different lockdown policies around the world in the pre-vaccine era, we reproduced a series of maps depicting the eight main lockdown policies… including school closures, workplace closures, the cancellation of public events, restrictions on gathering sizes, public transport closures, stay-at-home requirements, restrictions on internal movement, and restrictions on international travel (Figure 1). It is therefore not surprising that the effectiveness of lockdown policies has been under scrutiny in various studies.

Research has sought to define the concept of lockdown policies. Some scholars have conceptualised lockdown as a general status of nations [44] and cities [45] caused by the enforcement of several preventive measures. Others have enumerated the specific policy responses of different countries. For example, Djalante et al. conducted an analysis of pandemic responses in ASEAN (Association of Southeast Asian Nations) member countries and discussed the limits of regional cooperation in managing the crisis [25]. Other scholars have classified lockdown policies based on their level of stringency, such as soft lockdowns, moderate lockdowns, and hard lockdowns [32].

The literature has also highlighted the significance of lockdown policies and their influence on COVID-19 contamination. Stay-at-home policies are among the most frequently discussed measures in the literature. They have been considered to be the most conservative and effective anti-COVID-19 measures since the beginning of the pandemic, as such policies reduce social contact and maximise social distancing [18,20]. Travel restrictions constitute another important measure discussed in the literature. For example, the city of Wuhan successfully contained the pandemic through travel restrictions [22,29]. Estimates suggest that without the travel restrictions imposed during the early stage of the pandemic, the number of cases of COVID-19 would have been around 64% higher in the cities outside Hubei Province [29]. Studies have also found that restrictions on domestic and international mobility are associated with fewer COVID-19 cases and deaths [1,23]. Research has also examined other lockdown policies, such as restrictions on gatherings and border restrictions, demonstrating their positive effects with respect to preventing the spread of the pandemic [46,47].

School closures are yet another important response measure that has been found to contribute to a reduction in pandemic spread. For example, Viner et al. argued that school closures have been more effective in reducing the COVID-19 death rate than they were during for the SARS pandemic [30]. Other studies have also noted the positive effects of school closures with respect to controlling the pandemic [27]. However, some researchers note that school closures may have a counterproductive effect of COVID-19 contamination. Whereas school closures may increase community awareness of infection prevention, they are less effective than other government intervention measures in reducing the death toll due to the resulting possibility of increased family travel [18,28].

**Figure 2:**
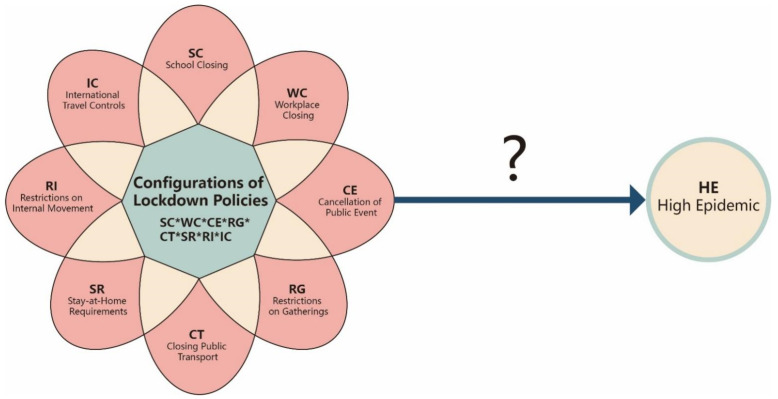
Conceptual framework of the present study. Note HE = high epidemic; SC = school closing (school closures); WC = workplace closing (workplace closures); CE = cancellation of public events; RG = restrictions on gatherings; CT = closing public transport (public transport closures); SR = stay-at-home requirements; RI = restrictions on internal movement; IC = international travel controls; * represents the intersection logic (i.e., AND).

In summary, whereas previous studies have examined the role of lockdown policies in containing the pandemic, the literature has not yet fully revealed the complex inter-relationships among different lockdown policies and their joint effects on COVID-19 containment. Research on the inter-relationships between lockdown policies and the COVID-19 pandemic has also yielded conflicting results. Such inconsistencies call for research that can explore the complexity of this issue. In this study, we apply qualitative comparative analysis (QCA) methodology, which is based on the configurational perspective, to offer theoretical and practical insights into the complex causal mechanisms and relationship between lockdown policies and the COVID-19 pandemic. The configurational perspective values the conjunctural effect of multiple conditions on an outcome, which can result in more profound insights in comparative policy studies [40]. In this research, we examine the complex causality (e.g., inconsistency and equivalence) between the configurations of conditions and the outcome (see Appendix A for a QCA-specific glossary). Therefore, this approach is appropriate for analysing the conjunctural effect of different lockdown policies on reducing pandemic spread [48]. Figure 2 presents the conceptual framework of this study. It consists of eight lockdown policies as the conditions: school closing (school closures), workplace closing (workplace closures), cancellation of public events, restrictions on gathering size, closing public transport (public transport closures), stay-at-home requirements, restrictions on internal movement, and restrictions on international travel. Each of these conditions represents a mainstream lockdown policy according to the Oxford COVID-19 Government Response Tracker [3]. The outcome is a high-epidemic situation of COVID-19.

## 3. Materials and Methods

### 3.1. Data Sources and Variables

We derive data for this study from the Oxford COVID-19 Government Response Tracker (OxCGRT) and the World Health Organization (WHO). The OxCGRT provides a systematic approach for tracking the policy responses to COVID-19 of governments around the world (see Appendix B for more information about OxCGRT). Given the difficulties stemming from the heterogeneity in terms of the actual responses of governments to COVID-19 and the diversity in terms of local contexts (e.g., political and sociocultural) in different countries around the world, the OxCGRT includes a series of indices that “provide a simple snapshot of the number and degree of government responses in a particular domain” [49]. Each index is calculated through an approach of composite measures, which combines a variety of indicators to provide an overall measure of the intensity of the focal policy. Therefore, such data from OxCGRT moves away from these nuances; they might leave out detailed information from a specific region/country (e.g., how well policies are enforced). However, this type of index approach enables efficient cross-national comparisons of government interventions [50], which is particularly suitable for the aim of our research. The value and quality of this data source is widely recognised by scholars [51,52,53].

In this paper, we selected the “containment and closure” portion of the OxCGRT system, which includes the indicators for the eight aforementioned types of lockdown policies, which we treat as the conditions. Specific explanations of these indicators are available at www.bsg.ox.ac.uk/covidtracker, accessed on 1 November 2021. In addition, to eliminate the influence of country size on the number of new infection cases, we chose the number of daily new infections per million inhabitants as a proxy for the outcome. As the effective period of lockdown policies is usually relatively long, the conditions of this study are relatively stable. This situation mitigates the risk of hysteresis between the conditions and the outcome, suggesting that the utilisation of cross-sectional data is acceptable if data collection is conducted at an appropriate point in time. We collected data as of 1 September 2020, which is an appropriate time for the following reasons: no globally recognized vaccine was available, no mass vaccination campaigns were taking place around the world, and lockdown policies were still the main approach for slowing the spread of COVID-19 [2]. Furthermore, as shown in the global mean index values for more than 180 countries over time [50], there were few variations in the lockdown index at that point in time among countries. It is also a time point at which the pandemic had spread for a sufficient period of time, making data monitoring and capture possible. As a result, this time point allows the study to cover several countries with complete data on the studied measures.

OxCGRT has been tracking government policy responses to COVID-19 in 186 countries around the world. We excluded those countries with incomplete data, as the QCA methodology does not allow for the processing of missing data. A representative sample of 84 representative case countries from six continents (Africa, Asia, Europe, North America, South America, and Oceania) are included in our analysis. The case selection process adheres to the principles of the QCA methodology [35].

### 3.2. Data Calibration

In fsQCA, each condition (i.e., each of the eight lockdown policies) and outcome (i.e., high-epidemic situations) is constructed as a fuzzy set, and each case (i.e., each country) is assigned a certain set membership score in each set. The process of assigning scores to cases is termed calibration [54]. Based on existing theoretical and empirical knowledge, we converted the data into the memberships of fuzzy sets through the indirect calibration method [55] (see Appendix C).

*Outcome*: According to the distribution of the adopted data (i.e., the number of daily new infections per million people), we chose the six-value scheme proposed by Ragin [56], in which 1 = fully in, 0.9 = mostly but not fully in, 0.6 = more or less in, 0.4 = more or less out, 0.1 = mostly but not fully out, and 0 = fully out.

*Conditions*: Because the scales of OxCGRT are all discrete sequenced data that have a clear progressive relationship with a floating range between 0 and 4 (see Table 1), we conducted equidistant calibration for each condition accordingly. More specifically, the maximum value in the coding instructions stands for “fully in”, whereas the minimum value represents “fully out”. For instance, in the row for school closing, the numeral “3” represents “fully in”, whereas the numeral “0” represents “fully out”. All of the terms in this terminology correspond to Ragin [35].

## 4. Results

### 4.1. Single-Condition Necessity Analysis

Consistent with mainstream QCA research, we first examined whether any single condition is necessary for the outcome [35], that is, whether any single lockdown policy can result in a high-epidemic situation (i.e., the outcome). Table 1 reports the results of this analysis for both conditions and their negations. The results show that no single lockdown policy on its own is necessary for the occurrence of a high-epidemic situation, as none of the consistency values exceeds 0.9. Thus, a high-epidemic situation is the result of the complex interactions among different conditions rather than a single condition [56]. Accordingly, we present the modelling and analysis determining the sufficiency of the configurations below.

### 4.2. Sufficiency Analysis of Condition Configuration

Based on the fsQCA modelling, we identified five pathways that lead to the occurrence of a high-epidemic situation under various lockdown policies (see Table 2). The overall consistency score is 0.762, which is above the minimum consistency threshold of 0.75. The overall coverage is 0.505. According to the core conditions in each configuration, they can be further classified into three patterns, namely “international travel, weak-confined”, “public transport, weak-confined”, and “school closing, weak-confined” (see Table 2). More specifically, the pattern of “international travel, weak-confined” consists of two pathways (i.e., P1a and P1b), whereas the pattern of “public transport, weak-confined” also consists of two pathways (i.e., P2a and P2b). One pathway (i.e., P3) is included in the pattern of “school closing, weak-confined”. Furthermore, all three configurations are higher than the lowest permitted value of 0.75. Interpretations of each configuration are presented below.

The “international travel, weak-confined” pattern (P1a and P1b): The core condition in the “international travel, weak-confined” pattern is ~international*gatherings. Despite the existence of restrictions on gatherings, the absence of international travel controls is the key condition that enables the occurrence of high-epidemic situations and is embodied in two pathways (P1a and P1b). In addition, there are several peripheral conditions exerting their influence on the occurrence of high epidemics in these two pathways. For P1a (workplace *publicevents *gatherings *~publictransport *~stayathome *~domestic *~international), the existence of workplace closures and the cancellation of public events play accessory roles, whereas the absence of these three conditions (i.e., public transport closures, stay-at-home requirements, and restrictions on internal movement) determine the formation of P1a. It should be noted that school closures have little influence on the formation of this pathway. The relevant countries include Albania, Slovenia, South Korea, and Serbia. For P1b (~school *workplace *publicevents *gatherings *publictransport *stayathome *domestic *~international), all of the lockdown policies other than its core conditions influence its formation by acting as peripheral conditions. The countries that are relevant to this pathway include Bolivia, Honduras, Iraq, Palestine, and Brazil.

The “public transport, weak-confined” pattern (P2a and P2b): With regard to the “public transport, weak-confined” pattern, the core condition of P2a and P2b is ~publictransport* domestic* international. Thus, under the combination of these conditions (i.e., the existence of international travel controls and restrictions on internal movement), the absence of public transport closures is the primary condition resulting in the occurrence of high epidemics. Furthermore, according to the components of P2a (i.e., ~school *workplace *publicevents *~publictransport *~stayathome *domestic *international), the combination of the existence of school closures, workplace closures, and the cancellation of public events, as well as the absence of stay-at-home requirements, acts as the set of peripheral conditions that supplements the formation of this pathway, along with the core condition. Relevant countries with respect to this pathway include Malaysia, Germany, Canada, Spain, Iran, and USA. The difference between P2b (~school *workplace *publicevents *gatherings *~publictransport *domestic *international) and P2a is that the absence of stay-at-home requirements is replaced by the existence of restrictions on gatherings within the configuration. Stay-at-home requirements are viewed as unnecessary in this pathway. This pathway has the largest number of case countries among all of the pathways. It includes Germany, Canada, Paraguay, Spain, Angola, Australia, Mexico, Kazakhstan, India, USA, and Panama.

The “school closing, weak-confined” pattern (P3): The “school closing, weak-confined” pattern consists of only one pathway, i.e., P3 (~school *workplace *publicevents *gatherings *~publictransport *~stayathome *~domestic). The combination of core conditions is ~school *workplace. This means that under circumstances wherein workplaces have been closed, the occurrence of a high-epidemic situation is mainly affected by whether or not schools remain open. The corresponding peripheral conditions are the existence of the cancellation of public events, the existence of restrictions on gatherings, the absence of closing of public transport, the absence of stay-at-home requirements, and the absence of restrictions on internal movement, whereas the existence of international travel controls plays a minimal role in this pattern. This pattern demonstrates the significance of the lockdown policy of school closures in limiting the spread of COVID-19, which is consistent with the prior literature [27,30]. The case countries under this combination of conditions are Slovenia, Switzerland, Belgium, Serbia, Portugal, and France.

## 5. Discussion

We found that both the existence and absence of international travel control measures can lead to a high-epidemic situation, depending on the configuration. For example, a high epidemic may occur in the absence of international travel controls in the P1a and P1b configurations or when international travel control policies are introduced in the P2a and P2b configurations. Configuration P3 further supports this assertation. Therefore, international travel control measures are not fully sufficient and must be combined with other lockdown policies to influence the outcome, corroborating several studies suggesting that international travel restrictions are the most effective in reducing pandemic spread when they are implemented in combination with other lockdown policies [57,58,59].

Although many countries in high-epidemic situations reduced international and domestic mobility (see Table 2), they did not implement public transport closures. In Table 3 (CT row), four of the five paths are represented by a white circle (i.e., condition is absent), whereas only P1b is represented by a dark circle (i.e., condition exists). Therefore, for CT, four paths demonstrate a “condition absence”, and only one path is “auxiliary”. As a result, we can conclude that the lack of attention to CT conditions is one of the most important factors leading to the occurrence of a high-epidemic situation. Various studies have found public transport to be an important vector of COVID-19 transmission. For example, an epidemiological study conducted in China by Hu et al. [60] showed significant risks of COVID-19 transmission among train passengers. Other studies have reported a significant correlation between the number of COVID-19 cases and the volume of domestic transportation, including trains and buses [61,62].

The results also emphasise the importance of school closures in controlling the spread of COVID-19. In line with the principles of the fcQCA methodology, Table 3 (ii) shows that SC and WC are the two most important lockdown policies in P3. The white and dark circles imply that when workplaces are closed but schools are not, a high-epidemic situation may still occur. In fact, several studies have emphasised the role of school closures in controlling epidemic spread [27,30]. Furthermore, as shown in Table 3 (ii), the absence of stay-at-home requirements (SR, indicated by the absence of a large circle, i.e., ⊙) across all of the pathways suggests that this policy has not been duly considered by governments and may have contributed to a high-epidemic situation in several countries. This finding demonstrates the significance of stay-at-home requirements for controlling epidemic spread, confirming the results of prior studies on the topic [18,20].

### Theoretical and Practical Implications

This study provides some important inferences useful for theory development. First, we note that no individual lockdown policy is necessary or sufficient to trigger a high-epidemic situation. The occurrence of a high-epidemic situation stems from a combination of multiple lockdown policies. The findings imply that different combinations of lockdown policies, which are the consequences of the different pathways representing the interactions among them, may all produce the same outcome, i.e., a high-epidemic situation. With this study, we identified three functional patterns, i.e., “international travel, weak-confined”, “public transport, weak-confined”, and “school closing, weak-confined”, which consist of five different pathways leading to the occurrence of a high-epidemic situation. Each pathway consists of different combinations of lockdown policies.

Second, this study demonstrates the importance and complexity of international travel restrictions. In previous literature reports, there has been a debate about the effect of international travel restrictions with respect to containing the spread of novel infectious diseases. For instance, Linka et al. [63] and Wells et al. [64] proved that international travel and border control was important for containing the pandemic, and Yang et al. [65] demonstrated that international travel control can delay local pandemic outbreaks for an average of five weeks. However, Errett et al. [66] and Abou-Setta [67] question the effectiveness of international travel control policies due to the difficulty of distinguishing the impact of travel bans from that of other epidemic lockdown measures. Our study advances our current understanding of the role of international travel controls; they are among the important measures that can reduce the spread of COVID-19, although their importance might be obscured when they are the only measure issued by governments. If an international travel control policy is not implemented in combination with other lockdown policies to control the spread of the virus, it can result in a high-epidemic situation. Such findings also correspond to several previous literature studies indicating that border closures are most effective when combined with other domestic control measures [59,68].

The third theoretical implication relates to mobility and public transportation. Transportation is one of the key enablers of mobility. Our study reveals that even when governments implement restrictions on international and domestic travel (i.e., ITC*RI), if there are no restrictions on public transportation (i.e., ~CT), a high-epidemic situation might still occur. Our study reaffirms the role that domestic mobility plays in the spread of COVID-19 [1,18,22,29]. Finally, the study reveals a complex inter-relationship between school and workplace closures. School closures remain a controversial measure with respect to reducing COVID-19 contamination [27,28,69]. This study provides new evidence on the relationship between school and workplace closure policies, suggesting that when workplaces are closed but schools remain open, a high-epidemic situation may still occur.

Practically, our findings suggest that no individual lockdown policy can mitigate the spread of the virus. The effectiveness of lockdown policies implemented by countries should be evaluated from a configurational perspective. Health policy makers should note that there are different patterns of high-epidemic occurrences consisting of various pathways of lockdown policy combinations. The same policy may function differently in different pathways, such as the “international travel control” and “school closing” policies. For example, when governments are considering whether or not to close schools, they need to carefully and holistically analyse the potential pathways to the occurrence of a high-epidemic situation by considering both the specific circumstances of their own countries and the lessons learned from other countries. This study also reveals that a common feature of high-epidemic countries is that they ignore the role of stay-at-home policies. Although the implementation of such policies is difficult, costly, and may undermine the governance system of several countries [70], stay-at-home policies play a significant role in mitigating the spread of the virus. China and Australia are examples of countries that were able to rapidly control the pandemic due to their stringent stay-at-home policies.

## 6. Conclusions

Many countries are still under the threat of the COVID-19 pandemic. In the pre-vaccination era, many countries relied on NPIs, such as lockdown policies, to reduce COVID-19 contamination. In this paper, we employed a configurational perspective based on the fsQCA methodology to evaluate the association between various lockdown policies and high-epidemic situations in 84 countries, highlighting the complex inter-relationships between different lockdown policies. The findings demonstrate that a combination of lockdown policies is more successful than any single lockdown policy, whereas the absence of several key measures in policy combinations can lead to a high-epidemic situation. The importance of international travel controls can become obscured when they are the only measure adopted, and a high-epidemic situation can still arise when restrictions are placed on international travel but not on public transport or when workplaces are closed but schools remain open. Theoretically, the elucidation and comparison of the complex configurations between lockdown policies and high-epidemic situations extends our knowledge of the significance of lockdown policies for controlling a highly contagious pandemic in the pre-vaccine era. The focus on this specific period is meaningful for both scholars and practitioners because the early phase of the pandemic is particularly significant [2].

These findings may serve as an empirical foundation for anti-pandemic policy making in the future, including policies targeting vaccine-resistant strains of COVID-19. The findings are also relevant to the formulation of COVID-19 containment policies in countries where vaccination has been limited to date. Such policies should also be informed by the adverse impacts of prolonged lockdown measures. Softer measures, such as the use of face coverings in addition to or instead of lockdown measures, should also be considered and tailored to the severity of virus transmission.

Despite the theoretical and practical value of the present research, it has certain limitations that readers should consider when using the findings. First, we investigated the combinations of lockdown policies on COVID-19 contamination across countries based on data from the OxCGRT. The data do not include very detailed information related to specific countries (e.g., the enforcement and implementation of government interventions and the local contextual characteristics that may affect epidemic spread). Hence, in this study, we did not consider variables such as the capacity of health services, population size, and percentage of the population over 65 years of age in the countries considered or other socio-cultural, political, and economic characteristics that have been shown to influence COVID-19 cases and deaths [1,23,24]. Although this is a common challenge faced by all domains of social science, it is important that future studies consider these variables in order to validate the stability and robustness of the results. In addition, future relevant research can extend perspectives beyond simply measuring the impact of policies; instead, it is necessary to incorporate the views of the general population of different countries with respect to lockdown measures, as policies are made to ensure public welfare. Future research should also include longitudinal studies to compare the relationship between NPIs and epidemic development using more significant time models. Obtaining more regular results could facilitate updates to anti-epidemic policies.

## Figures and Tables

**Table 1:** Necessity analysis of high-epidemic situations in the world.

Lockdown Policy	Consistency	Coverage
SC	0.831	0.547
~SC	0.373	0.484
WC	0.787	0.622
~WC	0.575	0.561
CE	0.815	0.513
~CE	0.204	0.405
RG	0.827	0.535
~RG	0.368	0.495
CT	0.534	0.676
~CT	0.602	0.402
SR	0.539	0.704
~SR	0.695	0.456
RI	0.654	0.545
~RI	0.455	0.417
IC	0.703	0.529
~IC	0.579	0.602

SC = school closing (school closures); WC = workplace closing (workplace closures); CE = cancellation of public events; RG = restrictions on gatherings; CT = closing public transport (public transport closures); SR = stay-at-home requirements; RI = restrictions on internal movement; IC = international travel controls; “~” represents “negation”, ~A = 1-A. Appendix A provides an explanation of the terms “consistency” and “coverage”.

**Table 2:** Consistency and coverage scores.

		RC	UC	C
	NC = f (SC, WC, CE, RG, CT, SR, RI, IC)			
*P1a*	WC*CE*RG*~CT*~SR*~RI*~IC	0.184	0.036	0.752
*P1b*	SC*WC*CE*RG*CT*SR*RI*~IC	0.178	0.102	0.859
*P2a*	SC*WC*CE*~CT*~SR*RI*IC	0.213	0.014	0.751
*P2b*	SC*WC*CE*RG*~CT*RI*IC	0.225	0.026	0.755
*P3*	~SC*WC*CE*RG*~CT*~SR*~RI	0.186	0.023	0.786
	solution coverage: 0.505			
	solution consistency: 0.762			

* represents the intersection logic (i.e., AND); “~” represents “negation”, i.e., the absence of a given condition.

**Table 3:** Configurations of pathways for high-epidemic situations.

	P1a	P1b	P2a	P2b	P3
SC		•	•	•	⊙
WC	•	•	•	•	●
CE	•	•	•	•	•
RG	●	●		•	•
CT	☉	•	⊙	⊙	☉
SR	☉	•	☉		☉
RI	☉	•	●	●	☉
IC	⊙	⊙	●	●	
Countries	ALB, SVN KOR, SRB	BOL, HND IRQ, PSE BRA	MYS, DEU CAN, ESP IRN, USA	DEU, CAN PRY, ESP AGO, AUS MEX, KAZ IND, USA PAN	SVN, CHE BEL, SRB PRT, FRA

Note: NC = new cases (per million); SC = school closing (school closures); WC = workplace closing (workplace closures); CE = cancellation of public events; RG = restrictions on gatherings; CT = closing public transport (public transport closures); SR = stay-at-home requirements; RI = restrictions on internal movement; IC = international travel controls; M = model; RC = raw coverage; UC = unique coverage; and C = consistency. “●/•” indicates the existence of the condition; the large circle represents the “core condition”, and the small circle represents the “peripheral condition”. “(⊙/☉/)” indicates the absence of a condition; the large circle is the “core condition”, and the small circle is the “peripheral condition”. Blank spaces indicate either presence or absence; “~” represents “negation”, ~A = 1-A. ALB = Albania; SVN = Slovenia; KOR = South Korea; SRB = Serbia; BOL = Bolivia; HND = Honduras; IRQ = Iraq; PSE = Palestine; BRA = Brazil; MYS = Malaysia; DEU = Germany; CAN = Canada; ESP = Spain; IRN = Iran; USA = United States; DEU = Germany; PRY = Paraguay; AGO = Angola; AUS = Australia; MEX = Mexico; KAZ = Kazakhstan; IND = India; PAN = Panama; CHE = Switzerland; BEL = Belgium; PRT = Portugal; FRA = France.

## Data Availability

Publicly available datasets were analyzed in this study. This data can be found here: [https://github.com/OxCGRT/covid-policy-tracker/tree/master/data].

## Appendix A. Introduction to Qualitative Comparative Analysis (QCA)

The basic logic of QCA is to explore the general characteristics of multiple cases by discussing the membership relationships between sets. In essence, it is a case-oriented rather than a variable-oriented research method, which can be applied not only in exploratory and inductive research but also in deductive research [35]. From the theoretical perspective, QCA can be used to validate, specify, and construct theories [31].

The steps for QCA implementation generally include the following: (1) The conditions and outcomes must be calibrated. Calibration is the process of assigning membership of data sets to cases, which is the basic premise for conducting QCA. (2) The necessity of a single condition is analysed. In QCA, if a certain (or some) condition(s) always exist(s) when a specific outcome occurs; this (or these) condition(s) can be viewed as the necessary condition(s) for the outcome. The “**consistency**” parameter is an important criterion to measure the necessary condition. When the consistency score of a certain condition is ≥0.9, it can be viewed as necessary for the outcome [35,54]. (3) The sufficiency of the condition configuration is analysed. In contrast to necessity analysis, the essence of QCA is to reveal the sufficient relationships between different condition configurations and outcome occurrence. As Schneider et al. consider that the consistency of sufficiency should not be less than 0.75 [54], in this paper, we set the consistency threshold to 0.75 and the frequency threshold to 2.

“**Coverage**” is another important criterion to assess the strength of the subset relations between sets and allows the strength of result interpretations carried out by different configurations to be compared through their coverage. Although there is no minimum threshold for coverage, lower coverage indicates that a given histology may be rarer [71]. There are various ways to calculate “coverage”. For instance, **raw coverage** (RC) measures the proportion of memberships in the outcome explained by each term of the solution. Raw coverage is computed for each solution term from the original data by dividing the sum of consistent membership in the solution term by the sum of the membership in the outcome. **Unique coverage** (UC) measures the proportion of memberships in the outcome as explained solely by each individual solution term (memberships that are not covered by other solution terms). This is computed by first removing the term from the solution and computing the solution coverage [34].

In addition, fsQCA can produce a complex solution, a parsimonious solution, and an intermediate solution for each analysis. The complex solution may result in overwhelming configurations and needless complexity due to the absence of a minimization process, which may jeopardize the subsequent pathway analysis. In contrast, the parsimonious solution allows for both easy counterfactuals and difficult counterfactuals during the minimization process, which can result in an unrealistically parsimonious interpretation and may neglect certain significant necessary conditions. Hence, Rihoux and Ragion suggest that an intermediate solution is superior to the other two solution types and should be a routine choice for any QCA research. Accordingly, in this paper, we chose to report the intermediate solution supplemented with the parsimonious solution [55]. This combination of two types of solutions allows the core conditions and peripheral conditions to be further distinguished; when a certain condition occurs in both the parsimonious solution and the intermediate solution, it is labelled as a core condition; if it only occurs in the intermediate solution, it is labelled as a peripheral condition. Furthermore, in this paper, we implemented the interpretation format of the model results proposed by Fiss [31]. A solid circle (●) represents that the existence of a condition, whereas a hollow circle (⊙) represents the absence (or negation) of a condition. A blank represents a fuzzy state, i.e., the condition may exist or be absent. A large circle represents a core condition (existing in both the parsimonious solution and the intermediate solution), whereas a small circle represents a peripheral condition (only existing in the intermediate solution).

There are several other QCA-specific terms that need be clarified. “**Causal complexity**” refers to a situation in which multiple explanatory factors of a phenomenon are combined in a complex and sometimes contradictory manner [72], i.e., there are multiple alternative paths to an outcome [33,73,74]. The QCA methodology is considered to be a very effective means of solving the problem of “causal complexity” [35,75,76].

The terms “**condition**” and “**outcome**” are specific to the QCA methodological system and correspond to the terms “independent variable” and “dependent variable” in traditional regression analysis.

## Appendix B. The Oxford COVID-19 Government Response Tracker (OxCGRT)

The OxCGRT tracks diverse policy responses in more than 180 countries around the world, with data collected from public sources by a team of more than 400 Oxford students and staff from around the world, covering the period from 1 January 2020 to present day. Through real-time collection and coding, the members of this large and diverse team use their background knowledge and expertise in 88 languages to parse reports and government announcements [50].

It should be noted that the responses of governments to COVID-19 exhibit significant nuance and heterogeneity. The effect of these diverse responses/policies is also inevitably influenced by local contexts (e.g., political and sociocultural factors). In order to enable systematic comparisons across countries, the OxCGRT project attempts to apply cross-national composite measures, which move away from these nuances by combining different indicators into a general index. To create this composite index, it applies a simple, additive, unweighted indexation approach that is relatively transparent and easy to interpret. The indices generated by this approach serve as baseline measures, which can be categorized into four policy indices, i.e., the overall government response index, stringency index, containment and health index, and economic support index. Each policy index is comprised of different combinations of policy indicators. To be more specific:

- **The government response index** was calculated using sixteen scaled indicators, including eight containment and closure policy indicators (i.e., school closures, workplace closures, the cancellation of public events, restrictions on gatherings, public transport closures, stay-at-home requirements, restrictions on internal movement, and international travel controls), two economic measure indicators (i.e., income support and debt/contract relief for households), and six health measure indicators (i.e., public information campaigns, testing policies, contact tracing, facial coverings, vaccination policies, and the protection of elderly people).
- **The containment and health index** was calculated using fourteen scaled indicators that largely overlapped with the government response index, with the exception of the two economic measure indicators.
- **The stringency index** was calculated using nine scaled indicators, including one health measure indicator (public information campaigns) and eight containment and closure policy indicators (the same indicators as those for the government response index and containment and health index).
- **The economic support index** was calculated by using two economic measure indicators, including income support and debt/contract relief for households.

More information about the definition of each indicator and the explanation and calculation of these indices can be found in Hale et al. [50]. In addition, the raw data are publicly available and can be downloaded for free at https://github.com/OxCGRT/covid-policy-tracker/tree/master/data, accessed on 1 June 2022.

It should be noted that the selection and measurement of the eight lockdown policy indicators is based on the OxCGRT project, and more details on data collection and disaggregation are available in Hale et al. [49].

## Appendix C. Calibration of Conditions and Outcome

**Table A1:** Calibration of Conditions and Outcome.

Outcome and Conditions	Description	Coding Instructions	Calibration
High epidemic	The number of daily new infections per million people	The numbers of daily new infections per million people in all case countries are standardized, ranging from 0 to 1	1 = fully in (100%); 0.9 = mostly but not fully in (90%); 0.6 = more or less in (60%); 0.4 = more or less out (40%); 0.1 = mostly but not fully out (10%); 0 = fully out (0%)
School closing	Recorded closings of schools and universities	0—No measures 1—Recommend closing 2—Require closing (only some levels or categories, e.g., just high school or just public schools) 3—Require closing all levels	3 = fully in (100%); 2 = more in than out (67%); 1 = more out than in (33%); 0 = fully out
Workplace closing	Recorded closings of workplaces	0—No measures 1—Recommend closing (or work from home) 2—Require closing (or work from home) for some sectors or categories of workers 3—Require closing (or work from home) all-but-essential workplaces (e.g., grocery stores, doctors)	3 = fully in (100%); 2 = more in than out (67%); 1 = more out than in (33%); 0 = fully out (0%)
Cancellation of public events	Recorded cancellations of public events	0—No measures 1—Recommend cancelling 2—Require cancelling	2 = fully in (100%); 1 = crossover (50%); 0 = fully out (0%)
Restrictions on gatherings	Recorded cut-off size for bans on private gatherings	0—No restrictions 1—Restrictions on very large gatherings (the limit is more than 1000 people) 2—Restrictions on gatherings of between 101 and 1000 people 3—Restrictions on gatherings of between 11 and 100 people 4—Restrictions on gatherings of 10 people or fewer	4 = fully in; 3 = more in than out (75%); 2 = crossover (50%); 1 = more out than in (25%); 0 = fully out (0%)
Closing public transport	Recorded closing of public transport	0—No measures 1—Recommend closing (or significantly reduced volume/route/means of transport available) 2—Require closing (or prohibit most citizens from using it)	2 = fully in (100%); 1 = crossover (50%); 0 = fully out (0%)
Stay-at-home requirements	Recorded orders to “shelter in place” and otherwise confine to home	0—No measures 1—Recommend not leaving house 2—Require not leaving house, with exceptions for daily exercise, grocery shopping, and ‘essential’ trips 3—Require not leaving house, with minimal exceptions (e.g., allowed to leave only once a week or only one person can leave at a time, etc.)	3 = fully in (100%); 2 = more in than out (67%); 1 = more out than in (33%); 0 = fully out (0%)
Restrictions on internal movement	Recorded restrictions on internal movement	0—No measures 1—Recommend not to travel between regions/cities 2—Internal movement restrictions in place	2 = fully in (100%); 1 = crossover (50%); 0 = fully out (0%)
International travel controls	Recorded restrictions on international travel	0—No measures 1—Screening 2—Quarantine arrivals from high-risk regions 3—Ban on arrivals from some regions 4—Ban on arrivals from all regions or total border closure	4 = fully in; 3 = more in than out (75%); 2 = crossover (50%); 1 = more out than in (25%); 0 = fully out (0%)

Note: The coding instructions for the eight lockdown policies are quoted from the Codebook for the Oxford COVID-19 Government Response Tracker (source: https://github.com/OxCGRT/covid-policy-tracker/blob/master/documentation/codebook.md, accessed on 1 November 2021).
